# Directional Change Mediates the Physiological Response to High-Intensity Shuttle Running in Professional Soccer Players

**DOI:** 10.3390/sports6020039

**Published:** 2018-04-24

**Authors:** Remy Tang, Conall Murtagh, Giles Warrington, Tim Cable, Oliver Morgan, Andrew O’Boyle, Darren Burgess, Ryland Morgans, Barry Drust

**Affiliations:** 1 School of Sport and Exercise Sciences, Liverpool John Moores University, Liverpool L3 3AF, UK; aoboyle@premierleague.com (A.O.); B.Drust@ljmu.ac.uk (B.D.); 2 Liverpool Football Club, Melwood training ground, Liverpool L12 8SY, UK; Conall.Murtagh@liverpoolfc.com (C.M.); Oliver.Morgan@liverpoolfc.com (O.M.); 3 Department of Physical Education and Sport Sciences, University of Limerick, Limerick V94 T9PX Ireland; giles.warrington@ul.ie; 4 Sport Science Department, Aspire Academy, P.O. Box 23833 Doha, Qatar; tim.cable@aspire.qa; 5 Arsenal Football Club, Arsenal training centre, London Colney AL2 1DR, UK; dburgess@arsenal.co.uk; 6 Everton Football Club, USM Finch Farm training ground, Liverpool L26 3UE, UK; ryland.morgans@evertonfc.com

**Keywords:** change of direction, deceleration, acceleration, accelerometry, fatigue

## Abstract

The purpose of this study was to investigate the influence that different frequencies of deceleration and acceleration actions had on the physiological demands in professional soccer players. Thirteen players were monitored via microelectromechanical devices during shuttle running protocols which involved one, three, or seven 180 degree directional changes. Heart rate exertion (HRE) (1.1 ± 0.7) and rating of perceived exertion (RPE) (5 ± 1) were significantly higher for the protocol which included seven directional changes when compared to the protocols which included one (HRE 0.5 ± 0.3, ES = 1.1, RPE 3 ± 0, ES = 2.7) or three (HRE 0.5 ± 0.2, ES = 1.1, RPE 3 ± 1, ES = 1.9) directional changes (*p* < 0.05). The gravitational force (g-force) as measured through accelerometry (ACC) also showed a similar trend when comparing the seven (8628.2 ± 1630.4 g) to the one (5888.6 ± 1159.1 g, ES = 1.9) or three (6526.9 ± 1257.6 g, ES = 1.4) directional change protocols (*p* < 0.05). The results of this study suggest that increasing the frequency of decelerations and accelerations at a high intensity running (HIR) speed alters the movement demands and elevates the physiological responses in professional players. This data has implications for the monitoring of physical performance and implementation of training drills.

## 1. Introduction

Soccer is a demanding sport with physical performance decreasing particularly after intense spells of activity and during the latter stages of a 90-min game [1,2]. The onset of fatigue during game-play is often determined by changes in HIR performance (moving at a speed greater than 5.5 m/s) [1,2,3]. Despite its popularity, the use of HIR as the sole marker of physical performance may be limited. For example, at a professional level, players cover an average HIR distance of 1151 m during a game which equates to approximately 10.6% of the total distance [4]. This relatively low percentage of HIR distance suggests that other factors, such as various game-specific movements, should also be considered when attempting to quantify physical performance in soccer. 

It is estimated that a change in activity occurs every 6 s during a 90-min professional game [5]. These changes include actions such as decelerations [6] and accelerations [7], which often occur at lower movement speeds. Research has indicated that professional soccer players complete, on average, 612 decelerations and 656 accelerations during a game [8]. The frequent occurrence of these actions justifies the need to further investigate how decelerations and accelerations may impact the physiological demands and potentially contribute to fatigue in professional soccer players. 

Studies which have looked at the physiological demands associated with deceleration and acceleration have commonly used directional change as a model. Directional change typically involves three different actions. These are, deceleration, turning, and acceleration. Studies which have attempted to evaluate directional change found that performing these actions caused an increase [9,10,11,12,13,14,15,16], decrease [17,18], or no change [19] in physiological response (e.g., oxygen consumption, blood lactate, heart rate (HR), RPE, etc.) when compared to protocols that involved constant speed running or less frequent changes in direction. The different outcomes of these investigations may in part be explained by the large variation in protocol design which tends to differ vastly between studies. This includes running speed, turning angle and intensity of deceleration/acceleration actions, etc. Further, research that has specifically looked at the physiological demands associated with performing different frequencies of directional change have utilised relatively slow movement speeds (a maximum speed of 4.4 m/s [13]) which, in turn, would have likely limited the intensity of deceleration and acceleration actions. Using an experimental design that applies a faster running speed would better replicate the higher intensity decelerations and accelerations that occur during soccer performance [8] and would provide some relevant new insight into the physiological demands associated with performing these types of actions. Such an approach may better explain physical performance in soccer and allow for a more comprehensive insight into the onset of fatigue.

The purpose of this study was therefore to design and evaluate three controlled intermittent high intensity exercise protocols that involve different frequencies of directional change in an attempt to quantify the physiological response associated with performing multiple deceleration and acceleration actions. The findings of this study should improve the understanding of the importance of decelerations and accelerations to the physiological response associated with HIR exercise protocols and provide some insight regarding the potential contribution that these actions have on fatigue in professional soccer players. 

## 2. Methods

### 2.1. Subjects

Thirteen male professional soccer players took part in the study. All subjects (age: 18 ± 1 years, weight: 79.5 ± 7.0 Kg, height: 1.8 ± 0.05 m) were playing at the under 18 or under 21 level for an English Premier League soccer club at the time of testing. All subjects were training and/or playing games at least five days per week consistently and had at least 12 months’ experience of being in a full-time training environment at a Premier League club. All subjects also had at least 12 months’ experience of providing subjective feedback associated with training load via the modified Borg Scale [20] and were familiar with wearing Global Positioning Systems (GPS) and HR monitors. The study was conducted according to the Declaration of Helsinki and was fully approved by the Liverpool John Moores University ethical board. Parental or guardian consent was obtained for any subjects under the age of eighteen. All subjects were assured that any involvement was voluntary and that they were free to withdraw from the study at any time.

### 2.2. Testing Procedures

The current investigation was a randomised controlled study that compared three testing protocols which involved different frequencies of directional change. To determine the physiological response associated with changes in the frequency of directional change, exercise variables which could influence the exercise response were controlled. These included the volume (i.e., total distance covered) and the running speed. Attempts were also made to ensure that the intensity of accelerations and decelerations during the protocols were matched through both audio/verbal feedback and the use of visual markings. Such an approach allowed for the independent variable in this study, directional change, to be isolated and its frequency manipulated accordingly. Accelerometry was used to monitor the impact of directional change and highlight any potential change in movement characteristics between protocols due to varying frequencies of deceleration and acceleration actions. Heart rate exertion and RPE were used to indicate the physiological and subjective response to the exercise challenge. 

### 2.3. Methodology

**Figure 1:**
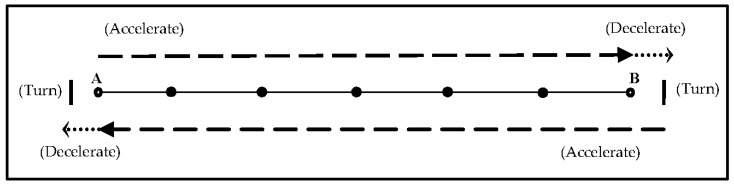
Diagrammatic representation of the testing protocols used. The example here illustrates the protocol used for the three directional changes (4 × 36 m). A similar approach was used for the 1 (2 × 72 m) and 7 (8 × 18 m) directional change protocols in the investigation. Briefly, subjects started at point A and were encouraged to accelerate as quickly as possible to the required running speed of 6 m/s moving towards point B. The target speed for each run was maintained using single repetitive audio signals and cones to help subjects judge their effort. Upon reaching the 36 m mark (point B) a different-coloured cone and hearing a double audio signal indicated the start of deceleration over a 2 m distance until reaching a clearly-marked line. Subjects then turned with both feet placed beyond the marked line and reaccelerated to the required running speed of 6 m/s taking them back to point A. Upon reaching point A subjects once again decelerated over a 2 m distance prior to turning. This process was repeated until the designated number of directional changes were completed.

All subjects (*n* = 13) completed at least one familiarisation session prior to testing to become acquainted with the key characteristics of the protocols (e.g., running speed and changing direction). The testing protocols required subjects to accelerate in a straight line to the required running speed of 6 m/s over distances of 72, 36, or 18 m. These distances were selected on a practical basis as they allowed for the running speed and total distance to be easily controlled across all three testing protocols. The total distance of each protocol was 144 m and involved subjects to complete one, three, or seven directional changes (see Table 1). Speed was controlled using a high pitch audio signal that played every second from an MP3 player (JVC, model NO.: CA-UXLP5). Cones were placed precisely 6 m apart along the track to enable subjects to evaluate their position and speed in conjunction with the audio signal. Subjects were encouraged to accelerate quickly at the start of each shuttle run to reach the required running speed as soon as possible. Video footage indicated that this was typically achieved somewhere between 6 and 12 m. Upon reaching the end of each shuttle run a different coloured cone and a double audio signal acted as an indicator for subjects to decelerate (over 2 m distance), stop, and turn with both feet placed behind a marked line (see Figure 1). The double audio signal was followed by a single audio signal after 1.5 s which marked the start of the second shuttle run, which began from the same cone which was initially used to indicate the start of deceleration. The 1.5 s delay was applied as pilot work using speed gates (Brower, Draper, UT, USA) indicated that this was the mean time it took for subjects to decelerate, turn, and return to the starting point for the second shuttle run. All testing protocols were carried out at the same location and on the same outdoor synthetic all-weather surface (Ligaturf 240 RS+, Burgheim, Germany).

All three testing sessions were at least one week apart and completed in-season between 9:30 and 10:30 am prior to training. Subjects were prohibited from taking part in any form of physical activity within the 48 h preceding testing. A 6-min standardised dynamic warm-up was common to all testing sessions. A rest period of at least 4 min between warm-up and data collection was also consistently applied. Once subjects had completed a testing protocol they were immediately seated on a bench located adjacent to the testing area for 5 min.

Microelectromechanical devices and subjective feedback were used on each testing day to determine the g-force, HIR distance, HR response, and the perceived exertion associated with each testing protocol. Each subject wore a 10 Hz GPS unit (Viper; Statsports, Newry, Ireland), which was positioned between the scapulae, using a tightly fitted, secured vest. Consideration of the limitations around the use of GPS when measuring high-intensity actions, such as accelerations and decelerations [21], resulted in the selection of data from the in-built accelerometer, which possesses a far higher sampling frequency. This data, sampled at 100 Hz, provided the g-force associated with each impact in the vertical, medial-lateral, and anterior-posterior plane. All the impacts recorded in each plane (negative entries made positive) were added to provide a total score which was then used to represent the total magnitude of g-force associated with each protocol. The raw data was accessed for analysis to reduce the influence of in-built algorithms affecting the data [22]. To standardise any potential error and maintain consistency throughout the data collection process each subject wore the same GPS unit for all testing protocols. High-intensity running distance was also collected through GPS for each directional change protocol. In the current study HIR was used as an indicator of the constant speed running distance covered at 6 m/s. 

Heart rate monitors (Polar T31, Kempele, Finland) were used to assess the physiological demands placed on subjects throughout each testing protocol. Heart rate exertion was used as an indicator of HR response for this investigation. This indicator was selected as it is one of the most common training load variables currently being used by professional soccer clubs [23] and provides a global index of the overall cardiovascular strain associated with an activity. Heart rate exertion is a weighted score, based on the subject’s HR with respect to their individual maximal heart rate (HR_max_) and represents the total volume of cardiovascular exertion subjects experience relative to time (see Equation (1)) [24]. Each subject’s HR_max_ was obtained by monitoring HR over a minimum duration of 12 months during competitive games, training sessions, and maximal endurance tests prior to the investigation. Maximal endurance tests were undertaken during pre-season, mid-season, and at the end of season. The highest HR_max_ score recorded for each subject during the 12 months preceding this investigation were used for testing. Where a new HR_max_ was recorded the HR curve was checked for potential error using appropriate software (Statsports, Ireland). Subject’s RPE was collected at five minutes after each testing session through the modified version of the Borg Scale [20]. This scale has been shown to be an effective method for collecting subjective feedback regarding the internal training load in soccer players post exercise [25]:

HRE = C·ΣW_i_·dt_i_ (1)

Equation (1): Heart rate exertion (Statsports, 2012), where i = 1 to n—the number of time points. C = scaling constant = 0.0167. W_i_ = HRE weighting for time point i based on HR/HR_max_ · dt_i_ = T_i_ − T_i−1_—time interval in seconds between successive HR values (0.1). n = number of HR values.

### 2.4. Statistical Analyses

Data were analysed using statistical software (SPSS version 21, Chicago, IL, USA). A one-way repeated measures ANOVA was used to determine the effect that each testing protocol had on the following dependent variables: g-force, HIR distance, HRE, and RPE. Where a main effect was found, a Bonferroni post hoc test was used to find where the significant differences occurred for each dependent variable. Effect sizes (ES) were calculated and its magnitude classified as trivial (<0.2), small (0.2–0.6), moderate (0.6–1.2), large (1.2–2.0), and very large (2.0–4.0) [26]. Confidence intervals (CI) set at 95% are also presented. The main-effect F values from the ANOVA were reported with the statistical significance set at *p* < 0.05. A Pearson’s correlation (significance set at *p* < 0.01) was used to assess the relationship between g-force and physiological response (HRE and RPE). This correlation analysis was repeated to also assess the relationship between HIR and physiological response (HRE and RPE). The criteria used for interpreting the magnitude of correlation were trivial (<0.1), small (0.1–0.3), moderate (0.3–0.5), large (0.5–0.7), very large (0.7–0.9), and almost perfect (0.9–1.0) [27]. Results are presented as means with standard deviations unless otherwise indicated.

## 3. Results

**Figure 2:**
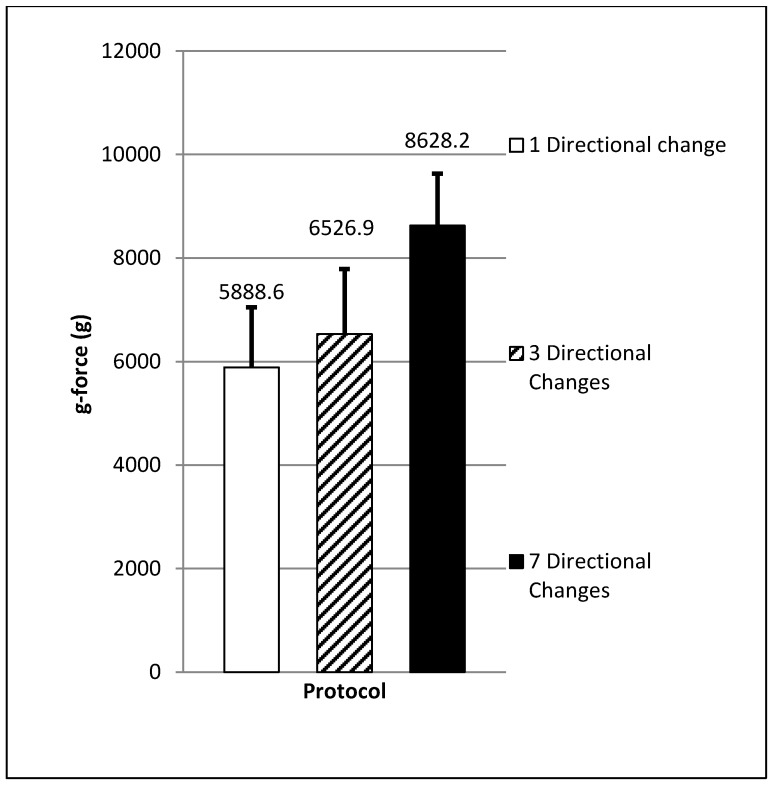
Mean gravitational force (*p* < 0.05; large ES).

**Figure 3:**
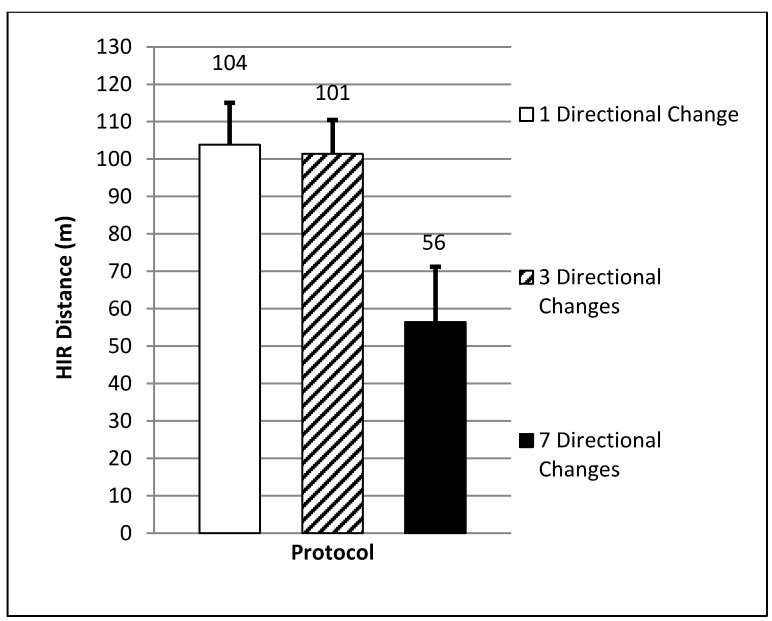
Mean high intensity running distance (*p* < 0.05; very large ES).

A significant difference in the mean total g-force (F = 496.50, *p* = 0.00) was found between the exercise protocols. This variable was significantly higher (24–32%) for the protocol that involved seven directional changes (8628.2 ± 1630.4 g, CI = 8308.7–8947.6) when compared to the protocol which included either one (5888.6 ± 1159.1 g, CI = 5643.0–6135.3, ES = 1.9 large, *p* = 0.00) or three (6526.9 ± 1257.6 g, CI = 6298.4–6755.4, ES = 1.4 large, *p* = 0.00) directional change(s) (see Figure 2). A significant difference in mean HIR distance (F = 88.49, *p* = 0.00) was also found when comparing the seven directional change protocol (56 ± 15 m, CI = 47–65) to the one (104 ± 11 m, CI = 97–111, ES = 3.53 very large, *p* = 0.00) or three (101 ± 9 m, CI = 96–107, ES = 3.52 very large, *p* = 0.00) directional change protocols (see Figure 3). Both protocols which involved the lowest number of directional change(s) (one and three) produced the highest mean HIR distance (72% and 70% of the total distance covered) when compared to the protocol which involved seven directional changes (HIR = 39% of the total distance covered). 

**Figure 4:**
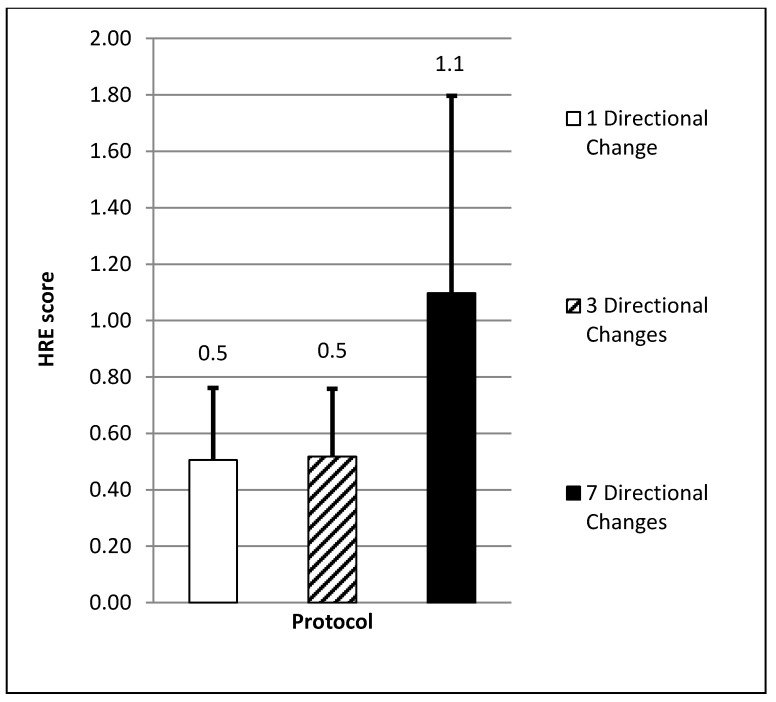
Mean heart rate exertion (*p* < 0.05; moderate ES).

**Figure 5:**
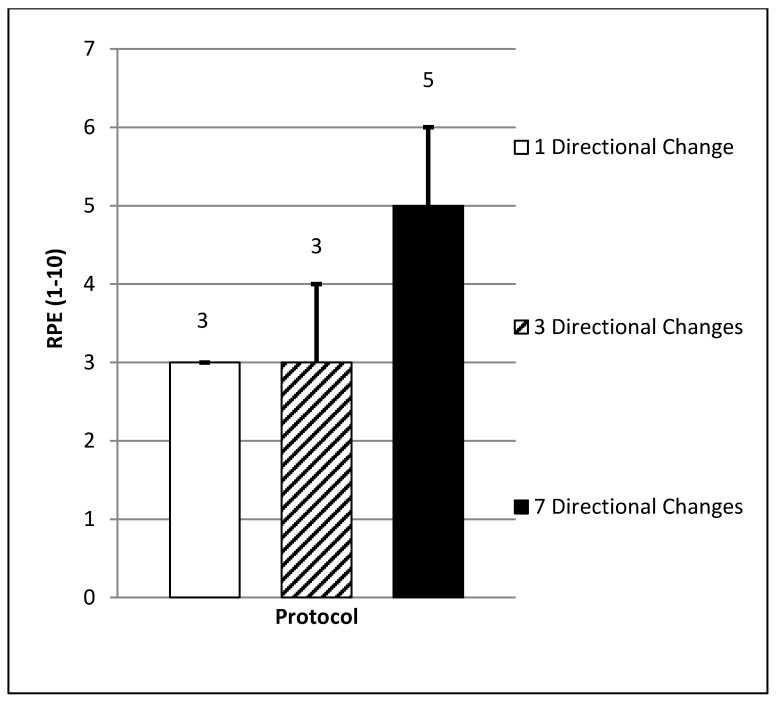
Mean rate of perceived exertion (*p* < 0.05; large to very large ES).

There was a significant difference in mean HRE (F = 11.47, *p* = 0.00) between the protocols. Subjects obtained the highest mean HRE during the protocol which included seven directional changes (1.1 ± 0.7, CI = 0.7–1.5). This value was found to be significantly higher (54.5%) than the protocols which involved either one (0.5 ± 0.3, CI = 0.4–0.7, ES = 1.1 moderate, *p* = 0.004) or three (0.5 ± 0.3, CI = 0.4–0.7, ES = 1.1 moderate, *p* = 0.005) directional change(s) (see Figure 4). A significant difference in mean RPE (F = 26.01, *p* = 0.00) was also found. The protocol which included seven directional changes resulted in the highest mean RPE score collected from all subjects (5 ± 1, CI = 4–5). This was significantly higher (40%) than the mean scores recorded in either the one (3 ± 0, CI = 3–3, ES = 2.7 very large, *p* = 0.00) or three (3 ± 1, CI = 3–4, ES = 1.9 large, *p* = 0.00) directional change protocols (*p* < 0.05) (see Figure 5). 

Correlations indicated that there was a significant positive relationship for total g-force when compared with both HRE (*r* = 0.45 moderate, *p* = 0.004) and RPE (*r* = 0.6 large, *p* = 0.000). In contrast to total g-force, HIR showed a significant negative relationship when compared with both HRE (*r* = −0.5 large, *p* = 0.001) and RPE (*r* = −0.7 large, *p* = 0.000).

## 4. Discussion and Conclusions

The current study represents a first attempt to show that frequent directional changes (decelerations and accelerations) performed at a HIR speed alters the “movement demands” and increases the cardiovascular and perceived exertion in professional soccer players. These findings suggest that such deceleration and acceleration actions carry the potential to increase player fatigue and, therefore, should be carefully considered when quantifying the physical performance in team sports such as soccer.

The current investigation is the first to show that a systematic increase in the frequency of deceleration and acceleration actions is related to a change in g-force during shuttle running. In contrast to previous research, which applied a lower running speed [10], our results clearly demonstrate a significant rise in g-force alongside an increase in the number of changes in direction. This increase could be due to the HIR speed applied in this study, which may have increased the intensity of decelerations and accelerations beyond those previously performed at lower speeds in other studies. The data collected in this study does not, however, allow us to confirm this suggestion as a range of running speeds were not investigated in our experimental design. The extent to which the increase in g-force observed here can be attributed to specific aspects (either deceleration or acceleration) associated with a change of direction is also unclear from our data. Previous research in soccer which looked at smaller turning angles concluded that this may be predominantly influenced by the deceleration phase of directional change [28]. Such findings, however, are not necessarily relevant to the 180 degree turns used in the current study which involved decelerating/ accelerating to/ from a stationary position.

An increase in g-force was accompanied by moderate increases in the cardiovascular demand and large increases in the subjective response to the exercise protocol. This data is supported by previous research which also observed an increase in HR and RPE due to an increase in frequency of directional change [10,14,16]. The physiological demands associated with changes of direction as isolated components (deceleration and acceleration) have not been adequately examined. For instance, no research has specifically looked at the physiological demands of deceleration. Although the literature would suggest that constant eccentric activity has a physiological cost that is almost twice lower than that seen with concentric activity [29,30,31,32], these findings are not necessarily relevant to performing short, intense, eccentric actions, such as the decelerations undertaken in the current investigation. To date, no studies have attempted to directly measure the physiological demands associated with acceleration in isolation, though some research does provide indirect attempts to calculate its physiological cost using equations which compare such actions to uphill running [33]. As decelerations and accelerations do not necessarily occur as part of directional change in professional soccer, there remains a need for research that utilises a direct approach when looking at the physiological responses associated with performing these actions in isolation. Heart rate exertion, the HR variable selected for this study, and commonly used to monitor the physiological response in professional soccer players [23], did carry some limitation within the current study design. As HRE is partly dependent on time, the different protocol durations would have affected the data from each trial. However, these differences in protocol duration would not account for the large mean HRE differences found within this study (HRE scores 54.5% greater for the seven directional change protocol when compared to the one or three directional changes).

Changes in movement demands as a result of changing direction were shown to positively correlate with physiological response. Correlations in this investigation showed moderate to high relationships between g-force and both the cardiovascular and subjective response to the exercise stress. Although further research is warranted, it may be that ACC could be an alternative indicator of intensity for drills incorporating deceleration and acceleration actions. High-intensity running distance was seen to have a large negative correlation with both HRE and RPE. This would suggest that total g-force, and not HIR distance, seemed to demonstrate a better relationship with the level of cardiovascular and subjective response to this particular exercise challenge. A possible explanation as to why the seven directional change protocol elicited a higher physiological response than the one and three directional change protocol could be related to the increased efficiency that may be associated with performing constant speed HIR at a speed of 6 m/s when compared to actions, such as intense decelerations and accelerations. Research suggests that more efficient mechanical energy stored in the series elastic component becomes available at speeds greater than 5 m/s [34]. It may, therefore, be that the exercise protocol that incorporated the most frequent directional changes (seven) resulted in less time running at a HIR speed and an increased time performing less energy-efficient movements (i.e., deceleration and acceleration) than the one and three directional change protocols. 

Coaches and practitioners should be aware that increasing the number of 180 degree directional changes (decelerations and accelerations) at a HIR speed elevates the cardiovascular and perceived demands in professional soccer players. The current study, therefore, highlights the potential importance of intense decelerations and accelerations when considering fatigue during game-play. An approach which considers these actions alongside HIR may provide a better representation of physical performance in professional soccer. The findings of this study also stress the importance of considering deceleration and acceleration actions when designing and implementing drills for training purposes. Although this study specifically looks at high-intensity shuttle running, its findings should be considered for any training drills which involve regular intense deceleration and acceleration actions. Such an approach should help to ensure the correct training stimulus is administered. Limitations of GPS in measuring deceleration and acceleration actions can make monitoring of training challenging. The results of the current study would suggest that g-force, as measured through microelectromechanical systems secured on the thoracic spine, which incorporate accelerometers, may be an alternative means of monitoring the intensity of such actions, although further research is warranted to confirm this. This study supports the need for a more holistic approach when quantifying physical performance and calls for a greater understanding of the various movement types and actions that may contribute to fatigue. 

In summary, this study attempted to isolate and manipulate the frequency of directional change in order to determine whether deceleration and acceleration actions could influence the cardiovascular and subjective response in professional soccer players. The data indicated that increasing the number of decelerations and accelerations, at a HIR speed, whilst controlling the distance and speed of movement, led to an elevated physiological response. Future research should focus on testing a range of faster running speeds whilst also attempting to isolate directional change into its components, in particular deceleration and acceleration, and systematically investigate the contribution of each individual action to the overall movement demand (g-force) and physiological response in professional soccer players.

## Figures and Tables

**Table 1:** Characteristics of the directional change (DC) testing protocols. Each protocol included one (2 × 72 m), three (4 × 36 m), or seven (8 × 18 m) 180-degree directional changes. The distance of each shuttle run decreased as the number of directional changes increased. The duration of each protocol also differed due to the 1.5 s delay allocated for each turn. All testing protocols had a total running distance of 144 m (excluding the 2 m deceleration zones).

	Total Running Distance	No. of Runs	Distance of Each Run	Protocol Duration	HIR Speed	No. of Decelerations	No. of Accelerations
1 DC	144 m	2	72 m	25.5 s	6 m/s	1	2
3 DC	144 m	4	36 m	28.5 s	6 m/s	3	4
7 DC	144 m	8	18 m	34.5 s	6 m/s	7	8
